# Interaction of caffeine with the SOS response pathway in *Escherichia coli*

**DOI:** 10.1186/s13099-015-0069-x

**Published:** 2015-08-18

**Authors:** Alyssa K Whitney, Tiffany L Weir

**Affiliations:** Department of Food Science and Human Nutrition, Colorado State University, 220 Gifford Building, Fort Collins, CO 80523 USA

**Keywords:** Caffeine, DNA repair, SOS response pathway, *Escherichia coli*

## Abstract

**Background:**

Previous studies have highlighted the antimicrobial activity of caffeine, both individually and in combination with other compounds. A proposed mechanism for caffeine’s antimicrobial effects is inhibition of bacterial DNA repair pathways. The current study examines the influence of sub-lethal caffeine levels on the growth and morphology of SOS response pathway mutants of *Escherichia coli*.

**Methods:**

Growth inhibition after treatment with caffeine and methyl methane sulfonate (MMS), a mutagenic agent, was determined for *E. coli* mutants lacking key genes in the SOS response pathway. The persistence of caffeine’s effects was explored by examining growth and morphology of caffeine and MMS-treated bacterial isolates in the absence of selective pressure.

**Results:**

Caffeine significantly reduced growth of *E. coli recA*- and *uvrA*-mutants treated with MMS. However, there was no significant difference in growth between *umuC*-isolates treated with MMS alone and MMS in combination with caffeine after 48 h of incubation. When *recA*-isolates from each treatment group were grown in untreated medium, bacterial isolates that had been exposed to MMS or MMS with caffeine showed increased growth relative to controls and caffeine-treated isolates. Morphologically*, recA*-isolates that had been treated with caffeine and both caffeine and MMS together had begun to display filamentous growth.

**Conclusions:**

Caffeine treatment further reduced growth of *recA*- and *uvrA*-mutants treated with MMS, despite a non-functional SOS response pathway. However, addition of caffeine had very little effect on MMS inhibition of *umuC*-mutants. Thus, growth inhibition of *E. coli* with caffeine treatment may be driven by caffeine interaction with UmuC, but also appears to induce damage by additional mechanisms as evidenced by the additive effects of caffeine in *recA*- and *uvrA*-mutants.

## Background

Caffeine is derived from plant leaves and is commonly found in consumed beverages such as tea and coffee at concentrations of 0.25–2.0% (w/v) [1]. Among the adult population in the United States, daily intake of caffeine is approximately 165 mg/day, largely through coffee consumption [2]. Due to its prevalence in the American diet, interactions between caffeine and pharmaceuticals likely occur, and there is value in exploring the interaction between caffeine and select antibiotics [3]. Previous studies have demonstrated that caffeine has direct antimicrobial activity, although reports of the specific inhibitory concentrations vary. Ibrahim et al. noted antimicrobial activity of caffeine against the human enteric pathogen, *Escherichia coli* O157 at concentrations as low as 0.5% (w/v)—similar in concentration to caffeine levels present in foods. This concentration resulted in slowed growth rate of *E. coli* O157 cells and reduced growth overall. However, at lower concentrations, caffeine is only bacteriostatic and *E. coli* growth recovered once the caffeine was removed [1]. Kang et al., reported failure of *E. coli* K12 strains to grow in concentrations of caffeine as low as 4 mg/mL (0.4% w/v) [3], but this increased sensitivity may be due to the differences between *E. coli* strains used in these studies.

Beyond bacteriostatic effects, caffeine synergistically enhances the activity of select antibiotics. Kanamycin activity is increased with the addition of 0.25 mg/mL (0.025% w/v) caffeine [3]. Kanamycin interacts with the 30 s subunit of prokaryotic ribosomes, inducing mistranslation of mRNA to protein [4] and is thought to damage DNA base pairs [3]. Thus, a potential target for caffeine activity is through interaction with DNA repair pathways, such as the SOS response pathway. Selby and Sancar observed increased sensitivity of bacterial cells to DNA damaging agents when pre-treated with caffeine [5]. Kang et al. noted that caffeine enhanced the sensitivity of specific mutants (*dnaQ, holC*, *holD* and *priA*) suggesting that caffeine acts by blocking DNA replication and causing double-strand breaks that require recombinatorial repair by *recA, recB, recC* and others [3]. Interestingly, the synergistic effect does not occur when caffeine is paired with bleomycin or cisplatin, and the activity of ciprofloxacin decreases when paired with caffeine [3]. Synergistic inhibition of bacterial growth in the presence of antibiotics and caffeine is therefore not ubiquitous, but rather depends on the mechanism of action of the antibiotic.

**Fig. 1:**
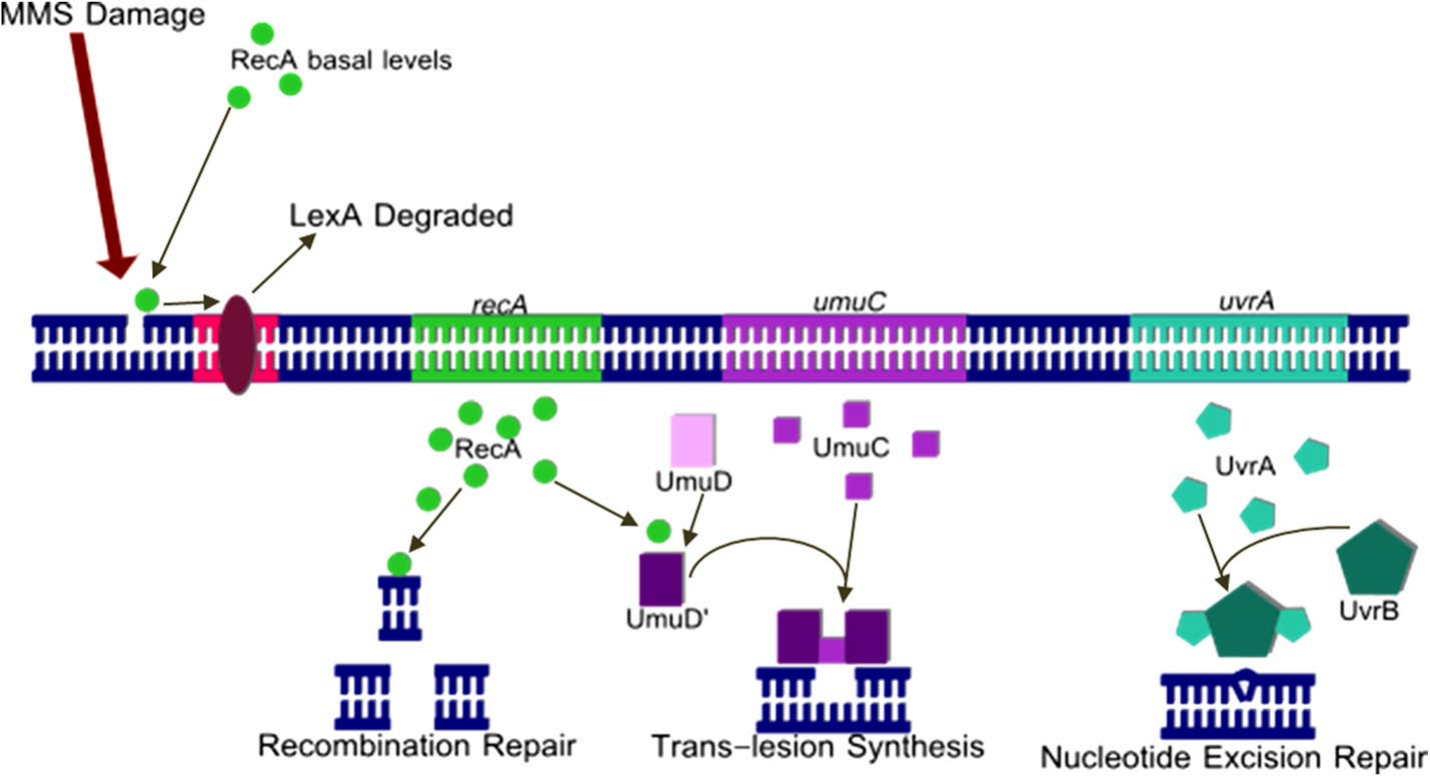
*E. coli* SOS response pathway. SOS response pathway in *E. coli* cells involving recombination repair (*recA*), trans-lesion synthesis (*umuC*), and nucleotide excision repair (*uvrA*).

The SOS response pathway is the bacterial DNA repair pathway activated in response to extensive damage to DNA, resulting in halted synthesis [6]. Several studies suggest that mutagen-potentiating effects of caffeine are mediated through interaction with DNA repair mechanisms, such as the SOS response pathway [7–9]. This hypothesis is supported through evidence of caffeine’s inhibition on SOS repair proteins in in vitro enzyme assays [10]. In the current study, we examine three of the genes regulated by the SOS pathway: *recA* (recombination repair)*, umuC* (trans-lesion synthesis), and *uvrA* (nucleotide excision repair) (Fig. 1).

Induction of the SOS response pathway begins when RecA protein, at basal levels in the cell, binds to single-stranded or double-stranded breaks in the DNA, becoming a co-protease that facilitates the degradation of LexA—a transcriptional repressor bound to the SOS box of the bacterial DNA [11]. Destruction of the repressor allows for expression of genes involved in DNA repair, marking the beginning of the SOS response pathway. The three genes of interest in this study are controlled by the SOS box in this fashion. RecA, responsible for the induction of the pathway [10, 12], encodes recombination repair, a mechanism that repairs double-stranded breaks and gaps in the DNA by using a template strand from a homologous DNA molecule [6]. UvrA, with UvrB and UvrC, is involved in nucleotide excision repair, the repair of mismatched nucleotides or dimers. Specifically, the A subunit aids in the delivery of the B subunit to the DNA where, along with UvrC, it removes damaged nucleotides and inserts the correct nucleotide based on the sequence of the complementary strand [6]. A previous study indicates the importance of this pathway in repair of damage induced by mutagenic agents like methyl methanesulfonate (MMS), an alkylating agent that leads to methylation of the DNA and induces single-stranded breaks [12]. UmuC, along with UmuD’, activated by RecA, form DNA Polymerase V, the molecule responsible for trans-lesion synthesis. This is generally regarded as the final step in the pathway. After approximately 40 min of stalled replication, DNA Polymerase V adds random nucleotides to the damaged region of the DNA to allow replication to continue [6]. This highly error-prone mechanism is known to be involved in repair of alkylation damage from MMS [12].

Activity of these three pathways is responsive to caffeine treatment. Recombination repair and trans-lesion synthesis are induced by caffeine [8], but are highly error prone and often introduce mutations to the DNA. Furthermore, the overall growth of cells deficient in RecA is reduced by 8 mM (0.15% w/v) caffeine, an inhibitory dose. In RecA deficient cells, this dose is less than the effective dose needed for inhibition of wild-type cells [13], suggesting caffeine sensitivity may be mediated through the recombination repair pathway. In enzyme assays, 10 mM (0.19% w/v) caffeine has been shown to inhibit the nucleotide excision repair pathway, potentially by interfering with the A subunit of the Uvr complex, as inhibition is greatest when caffeine is added to the reaction prior to the delivery of the B subunit to the damaged DNA [5]. After further study of this phenomenon, Sandlie et al. hypothesized that, by intercalating into the DNA and competing with the damaged DNA site, caffeine leads to nonspecific binding of the A subunit [7]. Thus, caffeine may be valuable in preventing recovery from mutagenic agents like MMS through interaction with the SOS response pathway, makes caffeine valuable for preventing recovery from mutation by other agents, like MMS [11]. Here we utilize MMS to induce DNA damage and activate RecA, nucleotide excision repair, and trans-lesion synthesis [12] in *E. coli* cells deficient in specific proteins involved in the SOS response, so we can examine the mechanism of action by caffeine in relation to SOS repair systems. We hypothesize that caffeine directly interferes with the SOS response in *E. coli* cells. Therefore, in strains with non-functional SOS response proteins, we expect to see no difference of *E. coli* growth between MMS with or without caffeine.

## Methods

### Bacterial strains

*E. coli* K-12 BW25113 parent strain and single gene deletion mutants *umuC*-JW1173-1, *recA*-JW2669-1, and *uvrA*-JW4019-2 were obtained from the Keio Knockout Collection [14], distributed by The Coli Genetic Stock Center at Yale University. All strains were cultured in Luria–Bertani (LB) growth medium at 37°C.

### Growth inhibition assay

**Fig. 2:**
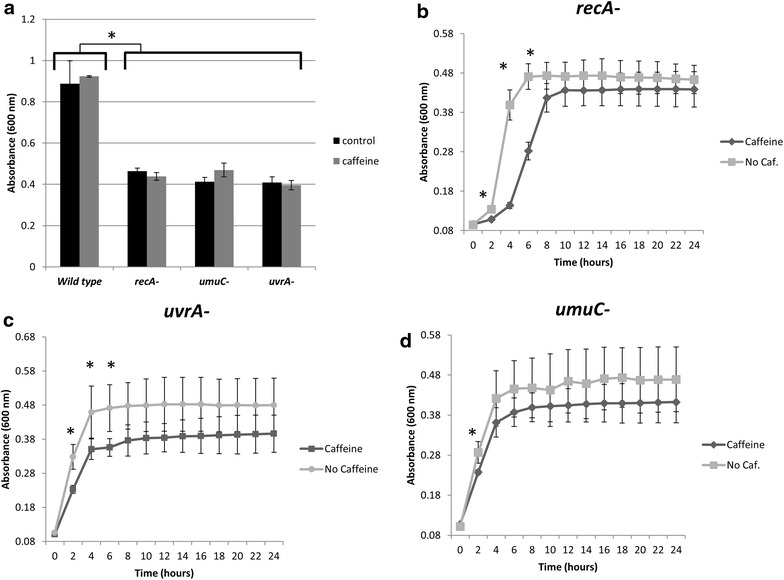
Caffeine effects on *E. coli Cells.* **a** Comparison of final cell density between untreated and caffeine treated (0.625 mg/mL) isolates of *E. coli* K12 wild-type and *recA*-, *uvrA*-, and *umu*C-mutants. **b** Represents the growth rate of *recA*-bacterial cells treated with caffeine alone, as compared to an untreated control. **c** Growth curve of *uvrA*-bacterial cells treated with 0.625 mg/mL caffeine alone, as compared to untreated control. **d** Growth curve of untreated *umuC*-mutants compared with cells treated with 0.625 mg/mL caffeine.

Bacterial isolates were chosen from LB plates and were then incubated at 37°C in LB broth (control and MMS treatments) or LB broth amended with 0.625 mg/mL (0.06% w/v) caffeine (MMS+ caffeine co-treatments) for 24 h prior to inhibition assays in a 96-well plate format. We demonstrated that this concentration of caffeine (0.625 mg/mL) was sub-inhibitory in parent *E. coli* strain (Fig. 2), and was lower than active concentrations demonstrated by Ibrahim et al. and Sandlie et al. [1, 6]. The optical density (OD_600_) of the overnight liquid cultures was measured by spectroscopy on a multi-mode plate reader (BioTek Synergy 2) and diluted in the same growth medium to OD_600_ = 0.02 prior to addition to assay plates.

Inhibition assays were conducted in 96-well plates, with each well filled with 100 µL of medium- LB broth for MMS alone and the control group, and LB broth amended with caffeine for co-treated wells. Controls were incubated in LB broth without added caffeine. 100 µL of 5 mg/mL MMS was added to the top row of each column in the treatment groups. In the control wells, the MMS was replaced with sterile water. The treatments and water control were diluted in a 1:2 serial dilution down the plate, with a final volume of 100 µL in each well. The final concentrations of MMS in the wells were 2.5 mg/mL (0.25% w/v), 1.25 mg/mL (0.125% w/v), 0.625 mg/mL (0.06% w/v), 0.313 mg/mL (0.0313% w/v), 0.156 mg/mL (0.0156% w/v), and 0.078 mg/mL (0.0078% w/v). The final concentration of 0.625 mg/mL caffeine remained constant throughout the caffeine treated wells. Assay plates were inoculated with 5 µL of diluted bacteria per well. The plates were covered with sealing film and placed into a BioTek reader programmed to incubate with shaking at 37°C and take measurements of absorbance at 600 nm every two hours. After completion of the assay, *E. colirecA*-cells were isolated from the 1.25 mg/mL MMS treatment wells (with and without caffeine) and preserved in 75% glycerol at −80°C for further experiments. The percent change in growth from the control was calculated using the following formula:

$$\% \;Change = \frac{{\left( {treatment - control} \right)}}{control} \times 100$$

where the control is equal to the average absorbance of the untreated wells and the treatment is equal to the average absorbance of the treatment replicates minus the absorbance of the MMS blank with the corresponding concentration. Each treatment was run in triplicate on a plate and each plate was repeated two times. Error bars were calculated from the standard deviation of the percent change in growth for each duplicate plate.

### Determination of treatment effects on growth and morphology of recA-bacterial isolates

To determine if the treatment conditions had persistent effects on cell growth and morphology, *recA*-cells that had been cryogenically persevered from control and treatment wells of the growth inhibition assays were grown on LB agar for 24–48 h. Single colonies were re-suspended in 500 µL LB broth and 2 µL from each culture were removed and added to 200 µL of untreated LB broth in a 96-well plate. The plate was incubated at 37°C with slow, continuous shaking and absorbance at 600 nm was measured every hour for an 8 h period. Three replicates of each treatment were tested.

Differences in cell morphology between treatment groups were examined by microscopy. As described, isolates of *E. colirecA*—from each treatment condition were removed from cryogenic storage and plated on LB agar for 24 h. Bacteria were suspended in 2–3 drops of water on slides and then dried and heat fixed. Cells were stained with a solution of 200 μg/mL Nile red in acetone and incubated at 37°C for 1 min in the dark and rinsed with dH_2_O. Slides were then destained in 70% EtOH and allowed to dry overnight. Slides were visualized under oil at 1,000× magnification on an Olympus BX3-CB11 microscope.

### Statistical analysis

Student’s T tests were performed in Excel to determine significant differences in growth inhibition between MMS and MMS-caffeine treated wells. Growth curve data were analyzed using 2-way ANOVA with post hoc Tukey’s test conducted with XL Stat-Pro. A 95% confidence interval was used, and values of P < 0.05 were considered significant.

## Results

### Effects of caffeine on *E. coli* SOS pathway mutants

To test the effects of caffeine on the SOS pathway, we confirmed that 0.625 mg/mL of caffeine, which was shown in previous experiments to be sub-inhibitory to several types of bacteria including, *E. coli* O157 (data not shown), did not inhibit the growth of wild-type *E. coli* K-12 or the mutant strains tested. After 24 h, there were no significant differences in cell density between treated and control isolates of wild-type or mutant *E. coli*; however, SOS mutants displayed significantly lower growth compared to the parent strain (Fig. 2a). This caffeine concentration was also incubated with each of the mutants to determine effects on growth rate (Fig. 2b–d). The growth rate of *E. coli recA*- and *uvrA*-cells was reduced, but the final cell density by 24 h was unaffected (Fig. 2b, c). Growth of *umuC*-isolates was initially delayed, but the differences between the treated and untreated cells resolved by 4 h and final cell density was not significantly different (Fig. 2d).

### Growth inhibition of *E. coli* SOS pathway mutants co-incubated with caffeine and MMS

**Fig. 3:**
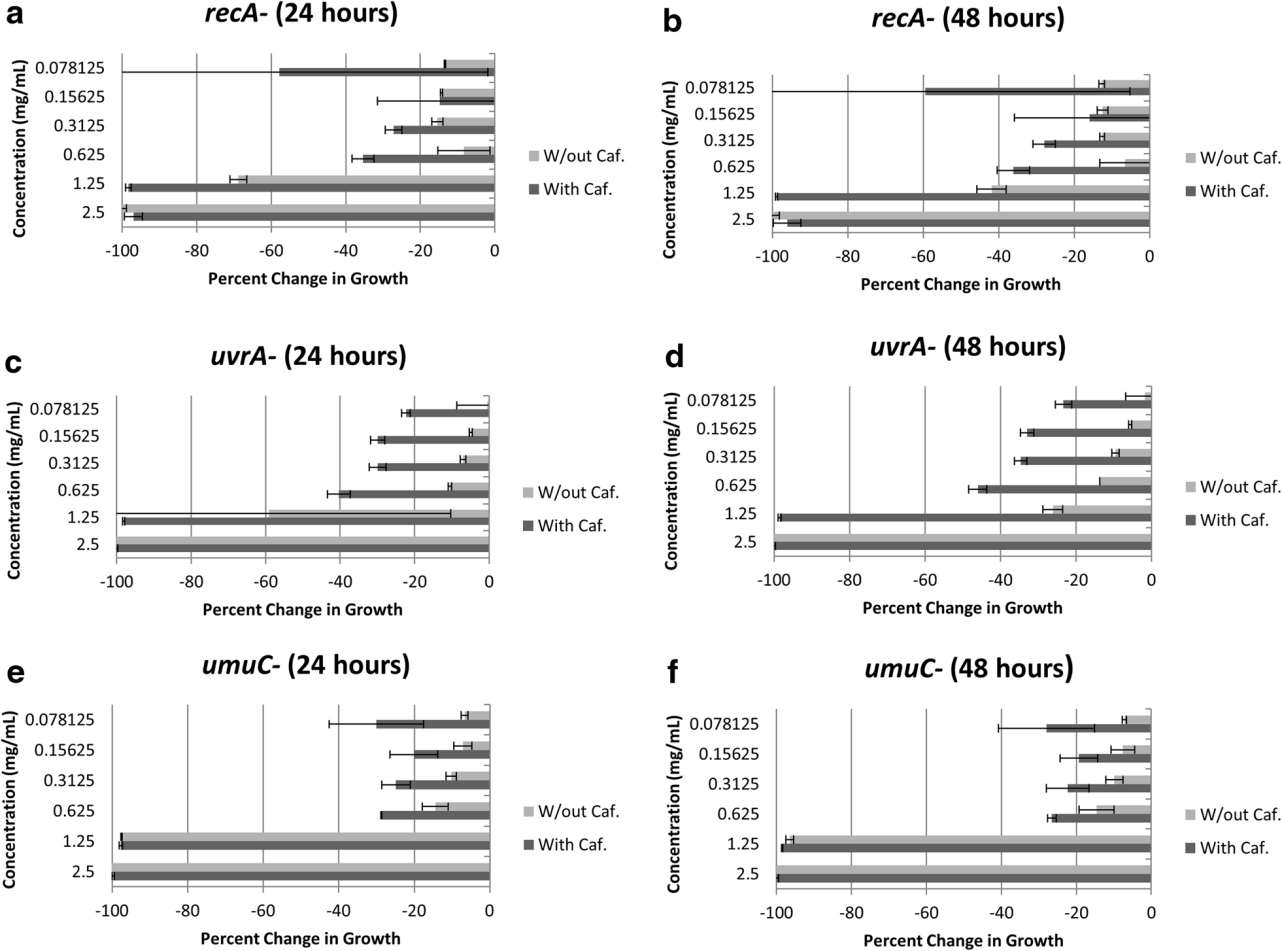
Growth inhibition of *E. coli recA*-cells. Change in growth of *E. coli* SOS pathway mutants when treated with 0.625 mg/mL of caffeine and varying concentrations of MMS, or MMS alone. **a** Represents density of *recA*-cells under both treatments at 24 h and **b** 48 h. **c** Growth inhibition of *uvrA*-cells under both treatments at 24 h and **d** 48 h. **e** Growth inhibition of *umuC*-cells treated with MMS with or without caffeine at 24 h and **f** 48 h. *Error bars* represent SE.

We examined the final cell density of *E. coli* SOS repair mutants after 24 and 48 h of exposure to MMS or caffeine and MMS combined. There was a significant potentiation of growth inhibition when *E. coli recA*- cells were co-treated with 0.625 mg/mL caffeine and several concentrations of MMS (1.25, 0.625, and 0.313 mg/mL), compared to treatment with MMS alone at the same concentrations (Fig. 3a, b). This increased inhibition with MMS and caffeine co-treatment was also observed in *uvrA*-cells. After 24 h, cells co-treated with 0.625 mg/mL caffeine and three concentrations of MMS (0.625, 0.313, and 0.156 mg/mL), showed decreased cell density compared to cells treated with MMS alone (Fig. 3c). Greater inhibition in co-treated cells relative to MMS treated cells persisted at 48 h with additional co-treatment concentrations (1.25 and 0.078 mg/mL MMS) showing significant separation at this time point (Fig. 3d). Unlike the *recA*- and *uvrA*-mutants, caffeine had little effect on *umuC*-cells treated with MMS. There was a small, but significant increase in growth inhibition of cells co-treated with 0.625 mg/mL caffeine and at MMS concentrations of 0.625 and 0.313 mg/mL compared to treatment with the same concentrations of MMS alone at 24 h (Fig. 3e). However, by 48 h, there was no significant difference in cell density of wells subject to caffeine and MMS co-treatment compared to MMS alone (Fig. 3f). In all mutants there was no additional inhibition by caffeine at the highest concentration of MMS as this concentration alone was sufficient to inhibit bacterial growth.

### Persistent effects of caffeine in recA-bacterial isolates

**Fig. 4:**
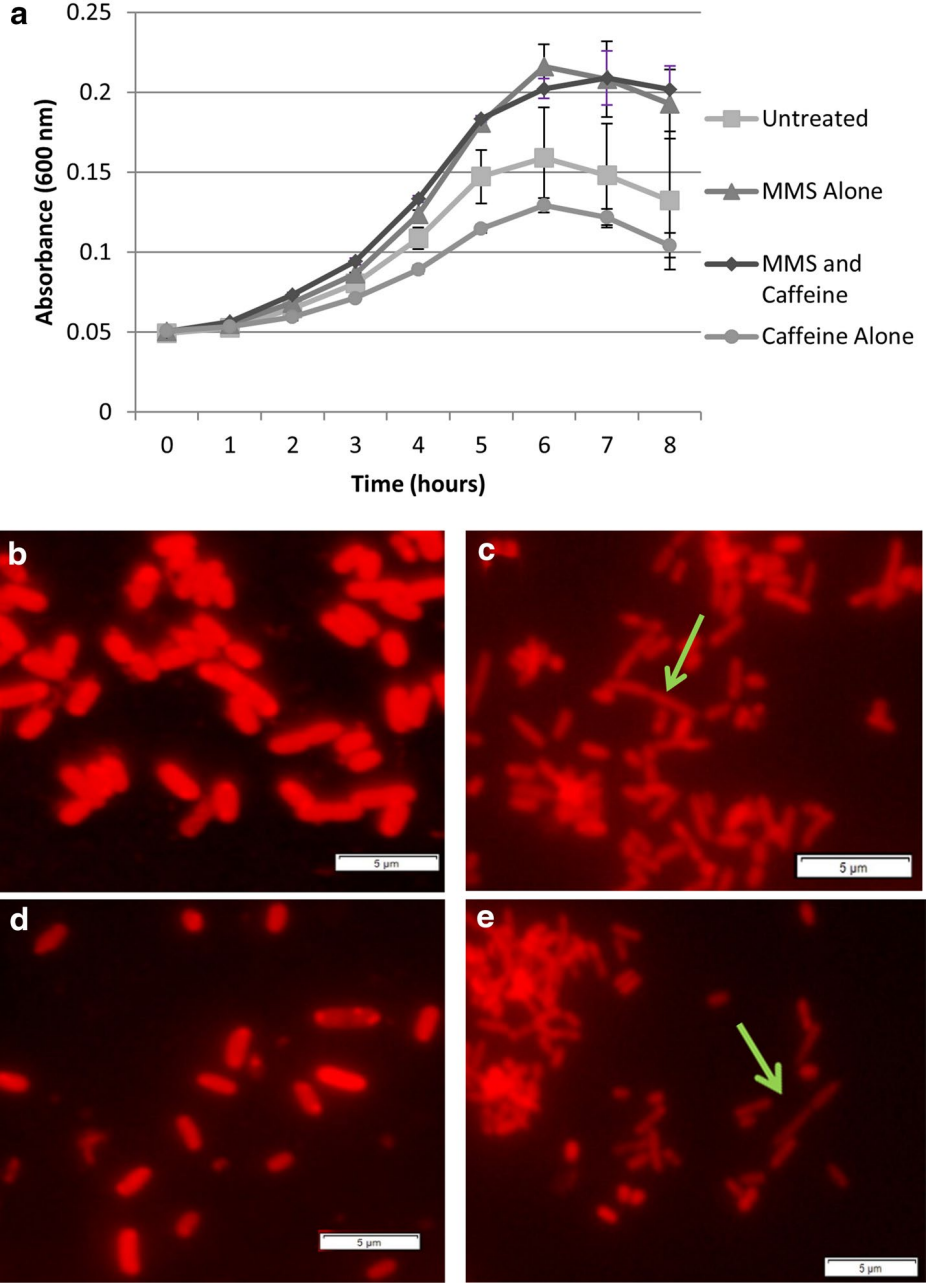
Persistence of caffeine effects on *recA*-cells. **a** Growth of *E. coli* JW2669-1 (*recA*-) when treated with 1.25 mg/mL MMS, 0.625 mg/mL caffeine, and MMS and caffeine co-treatment, in comparison with the growth of untreated cells. Morphology of *E. coli recA*-cells following inhibition assay. Treatment groups are no treatment (**b**), caffeine alone (**c**), MMS alone (**d**), and MMS and caffeine combined treatment (**e**). *Arrows* indicate cells displaying filamentous growth. Cells were observed at ×1,000 magnification.* Pictures scales* are 5 µm.

Because there was evidence of synergistic effects on growth of *E. coli recA*-cells when caffeine pre-treatment was combined with MMS, we explored the persistence of these effects in the absence of selective pressure. Cells isolated from wells treated with 0.625 mg/mL of caffeine and 1.25 mg/mL MMS, alone and in combination, and untreated wells were grown in LB liquid medium. Isolates from the MMS treatment groups (both with and without caffeine) displayed a higher growth rate and increased cell density after 8 h than isolates that had never been exposed to MMS (Fig. 4a). Although the isolates that had previously been exposed to caffeine and MMS grew significantly slower than those isolated from MMS treatment alone, final cell density at 8 h was not significantly different. Isolates that had been exposed to caffeine alone showed a reduced growth rate compared to control isolates, but by 8 h showed no significant differences in cell density from controls (Fig. 4a), which was consistent with the growth curves observed in *E. coli recA*-cells when caffeine was present (Fig. 2b).

We also examined cell morphology in *E. coli recA*-isolates after exposure to the various treatment conditions for evidence of filamentous growth, which is a known *E. coli* stress response [15]. Figure 4b represents the morphology of untreated *E. coli recA*-cells. After treatment with 0.625 mg/mL caffeine alone (Fig. 4c), the cells appear slightly smaller, and the beginning of filamentous growth is evident. After treatment with 1.25 mg/mL MMS alone (Fig. 4d), the cells appear unchanged in overall size, but there is some evidence of filamentous growth. After co-treatment with 0.625 mg/mL caffeine and 1.25 mg/mL MMS, there is clear evidence of filamentous growth in the *E. coli recA*-cells (Fig. 4e).

## Discussion

Most studies examining the effects of caffeine on the SOS pathway have not used sub-inhibitory caffeine concentrations, as was done in the current study (0.06% caffeine w/v). Therefore, it is difficult to compare the effects on *E. coli* growth that we observed with those previously reported. This study is among the first to demonstrate a reduction in growth rate due to caffeine exposure, but without effects on final cell density achieved as cells entered stationary phase. In the current study, caffeine significantly potentiated the activity of MMS when added as a pretreatment to *recA*- and *uvrA*-mutants. The synergistic interaction seen with co-treatment suggests that caffeine does not inhibit *E. coli* cell growth through interaction with RecA (recombination repair) or UvrA (nucleotide excision repair), two important proteins in the SOS response pathway. Obana et al. reported a failure to significantly reduce SOS functioning with caffeine treatment and noted no difference in SOS activity level with caffeinated and decaffeinated coffee [10]; a result that was consistent with the lack of direct influence observed here. Although our data indicate that caffeine does not interact with RecA or UvrA, there are synergistic interactions between caffeine and MMS, suggesting further study of caffeine’s inhibitory mechanisms are warranted as there is potential value for caffeine as an antibiotic adjuvant.

Caffeine may affect cell growth by inhibiting UmuC and subsequently, DNA Polymerase V and trans-lesion synthesis. When added alone, caffeine had almost no effect on the growth rate of *umuC*-cells compared to the other mutants tested (Fig. 2d). Likewise, we observed little significant difference between treatment with MMS alone and co-treatment with MMS and 0.625 mg/mL caffeine after 24 h and no significant difference after 48 h. In a knockout *E. coli* strain where no UmuC is produced, there would be no expected difference in growth between MMS and caffeine/MMS co-treatment. Kim and Levin indicated possible interaction between caffeine and UmuC in their 1990 study [8], but this topic has been largely unstudied in the intervening time, and further research is required to fully discern the effect of caffeine on UmuC.

Finally, we examined the growth and cellular morphology of *E. coli recA*-cells isolated from assay treatments in the absence of selective pressure to explore the persistence of caffeine’s effects. Surprisingly, the cells that had been isolated from MMS and caffeine/MMS co-treatments had a higher growth rate and final cell density compared to untreated cells and those treated only with caffeine (Fig. 4a). This response is likely due to activation of an RpoS-mediated stress response following MMS exposure and cryogenic storage of the cells. This is a commonly triggered stress response in *E. coli* and related bacteria which results in rapid accumulation of the alternative sigma factor RpoS, and activation of approximately 500 genes that render cells more resistant to generalized stress conditions [16]. Interestingly, caffeine treatment alone did not result in the same accelerated growth when stress was removed, but isolates previously exposed to caffeine behaved similar to when caffeine was present in the growth medium, indicating that caffeine exposure caused persistent damage. When we examined these isolates under a microscope, we also observed some filamentous growth in caffeine treated cells. Filamentous growth is a known *E. coli* stress response initiated by DNA damage and activation of SOS responses [15] and allows the cells to continue proliferating at the same rate, although they do not fully divide. Thus, it is likely that caffeine is independently causing damage to the DNA and triggering filamentous growth.

In addition to direct interaction with SOS pathway proteins, several other mechanisms have been proposed to explain caffeine’s inhibitory effects on bacterial cell growth. These include inhibition of protein synthesis and interference with ATP binding [17]. There is also a report of direct interaction with MMS, potentially through dimer formation [18]. However, there are few conclusive studies investigating these effects. Intercalation of caffeine in DNA is also believed to be responsible for the observed inhibition of *E. coli*. Caffeine is shown to be a DNA intercalating agent, largely due to similarity in structure to purine nucleotides, and may lead to non-specific binding of repair proteins in the cell [3, 5, 13], rather than directly interacting with the proteins themselves. Intercalation may also lead to double-stranded breaks in the DNA and frameshift mutations. Furthermore, the increased stress of intercalation by caffeine, in addition to alkylation stress from MMS, could result in observed synergistic interactions and filamentous growth, making the cells more vulnerable to further damage under selective pressure.

## Conclusions

Caffeine has existing medical relevance as an adjuvant in many over-the-counter painkillers [19]. Previous studies have indicated synergistic interaction of caffeine with kanamycin [3] and DNA damaging agents [5]. Identifying the cellular targets of caffeine helps determine which antibiotics would be most effective in combination, as not all antibiotics interact with caffeine in a synergistic manner [3]. We determined that inhibitory activity of caffeine against *E. coli* K12 strains is probably not due to interaction with RecA and UvrA proteins, but may occur through interaction with UmuC and the inhibition of trans-lesion synthesis. Furthermore, observed synergistic interactions of MMS and caffeine may be due to caffeine intercalation in addition to alkylation stress of MMS to bacterial DNA, unrepairable by trans-lesion synthesis, the final step of the SOS response pathway. This DNA damage leads to filamentous growth of *E. coli* cells, allowing the bacteria to continue proliferation but without full cell division. Filamentous growth makes the bacteria more vulnerable to further damage by the MMS and caffeine, because the lack of septa between cells facilitates the diffusion of small molecules. Reduction of *E. coli* growth by caffeine in a synergistic fashion lends support toward the use of caffeine as an antibiotic adjuvant.

## Abbreviations

w/v: weight/volume
MMS: methylmethane sulfonate
DIC: differential interference contrast
